# Delayed disengagement of attention from snakes in children with autism

**DOI:** 10.3389/fpsyg.2015.00241

**Published:** 2015-03-03

**Authors:** Tomoko Isomura, Shino Ogawa, Masahiro Shibasaki, Nobuo Masataka

**Affiliations:** Primate Research Institute, Kyoto University, InuyamaJapan

**Keywords:** autism, snake fear, attention, cognitive impairment, biological adaptation

## Abstract

In the visual search task, it is well known that detection of a tilted straight line as the target among vertical lines that act as distractors is easier than vice versa, and that detection of a snake image as the target among flower images is easier than vice versa. In this study, the degree of such search asymmetry was compared between 18 children with autism and 14 typically developing (TD) children. The results revealed that compared to TD children, children with autism were disproportionally slow when asked to detect the flower among the snake images, suggesting the possibility that they experienced difficulty of disengaging their attention from the snake images. This delayed disengagement would serve itself as an enhanced attentional bias toward snakes in children with autism that is similar to characteristics of visual search performance in anxiety patients.

## INTRODUCTION

Particular characteristics of childhood autism involve a profound impairment of communication (American Psychiatric Association, 2013). Typically, children with autism are known not to pay attention to things on demand, for example, when things are pointed to by caregivers, when they are called by name, or when someone enters the room (Markram and Markram, 2010 for review). It is in fact notoriously difficult to engage their attention on demand (Johnson et al., 1993). Nevertheless, they are also well known to pay abnormal and obsessive attention to detail, and to note and record their environment with exquisite clarity (Casey et al., 1993). They are capable of becoming hyper-focused and locked-in on apparently arbitrary subjects of interest, and of sustaining their attention on these subjects for unusually long periods of time. So far, these seemingly conflicting phenomena regarding the characteristics of attention impairment in autism have been interpreted as consequences of excessive on-going processing and excessive attention to endogenous domains where attention is fed back onto oneself; as a result of this internal hyper-focus, it would be more difficult for another person to command the attention of the child with autism, and it would also be more difficult for the child himself/herself to command his/her own attention voluntarily (Posner and Dehaene, 1994). This explanation is indeed confirmed by findings about the impairment in disengaging and shifting attention in children with autism (Hughes and Russell, 1993; Van Der Geest et al., 2001; Landry and Bryson, 2004; Elsabbagh et al., 2009), who on the other hand have been reported to behave comparably to typically developing (TD) children with respect to visual orienting performance *per se*. While all these studies were conducted by presenting biologically neutral, simple stimuli such as purely geometric shapes to the participants, the present experiment was conducted by presenting snake images as evolutionally relevant threatening stimuli (Isbell, 2009).

So far, three recent publications have shown that delayed disengagement of attention occurs in adults (Fox et al., 2002; Belopolsky et al., 2011) as well as children (Yorzinski et al., 2014; without any developmental disorder) robustly in association with complex social and biological stimuli associated with threat, but no such study has been undertaken in children with autism. Meanwhile, a series of investigations has shown that human children as well as adults have an attentional bias toward the detection of fear-relevant stimuli, such as snakes, compared to neutral stimuli, such as flowers (Öhman and Soares, 1993; Öhman and Mineka, 2001; Öhman et al., 2001; Flykt, 2005). In these studies, participants are typically presented with 3-by-3 matrices of fear-relevant and neutral images. The images are presented either in black and white or in colour, and regardless of color content, reaction times (RTs) have been found to be significantly shorter for fear-relevant targets than for neutral targets. Recent studies have documented that preschool children, 8- to 14-month-old infants, and even non-human primates also detect snakes more quickly than flowers in gray-scale (LoBue and DeLoache, 2008; Shibasaki and Kawai, 2009; LoBue and DeLoache, 2010; Masataka et al., 2010; Hayakawa et al., 2011).

In the present study, we hypothesized that snake images would influence the visual search performance of children with autism differently from that of TD children. Namely, when an image of a flower was presented as the target with images of snakes as distractors, children with autism would show more difficulty of detecting the target than TD children because of the slower disengagement from the threatening stimuli. If the results of the analysis of the collected data confirmed this prediction, it would indicate that the delay would associate with enhanced phobia and anxiety levels that are prominent in this developmental disorder (Markram and Markram, 2010). To evaluate the effects of such threatening stimuli, another pair of search tasks using non-biological, purely geometric items was administered; these geometric images were distinguished from one another by a feature difference along a single dimension (tilted vs. vertical straight lines). In the present experiments, all conditions were tested separately over a total of four blocks (each of four different types of images as a target) by requiring participants to touch the target image presented on a touch-pad.

## MATERIALS AND METHODS

### ETHICAL STATEMENTS

This investigation was conducted according to the principles expressed in the Declaration of Helsinki. All experimental protocols are consistent with the Guide for Experimentation with Humans and were approved by the Institutional Ethics Committee of the Primate Research Institute, Kyoto University. We obtained written informed consent from the parents of all participants involved in our study.

### PARTICIPANTS

Two groups of 8- to 10-year-old children participated: a group of 18 children who had been diagnosed as meeting DSM-5 diagnostic criteria for autism (American Psychiatric Association, 2013) without any anxiety or phobic symptom by psychiatrists from several hospitals in Kyoto Prefecture, Japan, and a group of 14 TD children in Japan. All children with autism and all TD children were assessed using the Autism Spectrum Quotient (Auyeung et al., 2008) at the commencement of the present study. The maximum score reported in the TD group was 17, whereas the minimum score reported in the group of children with autism was 24. The cognitive ability of the participants was assessed using WISC III, Raven’s Progressive Matrices, and Picture Vocabulary Test. Scores on each test, as well as the mean chronological age, did not differ significantly between the groups (**Table 1**).

**Table 1:** Chronological ages (years:months), WISC IQ Scores, Raven’s Matrices raw scores, Picture Vocabulary Test (PVT) scores, Autism-Spectrum Quotient (ASQ) for the participants*.

Participant	With autism (*N =* 18)	Control (*N =* 14)	Significance of difference (*p*)**
Age	9:3 (0:79)	9:3 (0:94)	0.975
WISC III			
Verbal IQ	101.7 (16.59)	103.6 (17.44)	0.752
Performance IQ	98.1 (13.59)	101.8 (15.90)	0.475
Full-Scale IQ	100.0 (14.84)	105.6 (15.45)	0.602
Raven’s Matrices	29.4 (4.03)	29.4 (4.53)	0.994
PVT	52.4 (11.45)	52.2 (11.65)	0.974
ASQ	29.1 (5.55)	11.71 (4.36)	< 0.000

*Scores presented are mean values (SDs).

**Probabilities were evaluated by unpaired *t*-tests (df = 30).

### MATERIALS AND PROCEDURE

Each participant underwent two different visual search experiments in this study. Each of the experiments again involved two search tasks. A touch-screen monitor was used for the stimulus presentation. For the first experiment, we selected 48 grayscale photographs; half of the images depicted flowers and half depicted snakes. In a given trial, nine of these photographs were displayed in a 3-by-3 matrix. Each image matrix was presented on a 38.1-cm (15-inch) screen of the monitor. Each matrix contained one target image from one category and eight distractor images from the other category. This yielded two combinations: a snake among flowers, and a flower among snakes. Each of the 24 images in the target category served as the target once. Each of the 24 pictures in the distracter category appeared eight times on average; the different distracters were presented approximately the same number of times across trials. The stimulus order was created by randomly arranging the matrices.

In both tasks of the second experiment, the stimuli consisted of two possible items that were distinguished from one another by a feature difference along a single dimension. The distinguishing feature was whether a given 0.7-cm-long straight line was tilted (rotated 18^∘^ counterclockwise) or vertical. In one task, the target was a tilted line that was presented among 11 vertical line distractors. In the other task, a vertical line was the target, and the distractors were 11 tilted lines. In each task, 36 different patterns of stimuli were presented. Stimulus displays consisted of 12 elements (i.e., straight lines) arranged around an imaginary 6.2-cm by 8.4-cm square centerd around a fixation point. Each element measured 0.7-cm by 0.7-cm, subtending at a visual angle of approximately 1.0^∘^ horizontally and 1.0^∘^ vertically. The minimum distance between the centers of each element in any display were 1.2-cm between positions in a row and 1.2-cm between positions in a column, and the items were presented in random locations across the screen.

In each experiment, the participant was seated in front of the monitor (approximately 40 cm from the base of the screen) and was told to place his/her hands on the same place at the start of each trial, making it possible to collect reliable latency data. An experimenter was seated next to the participant to monitor and instruct the child throughout the procedure.

A set of nine practice trials was completed at the beginning of the first experiment to instruct the child on how to use the touch screen. In the first three trials, a display consisting of one target (an image of a puppet) and eight distracters (images of another puppet) was presented. The participant was asked to touch the target among distracters as quickly as possible and then return his/her hands to the handprints. In the next six trials, the display consisted of one target (a snake or a flower) and eight distracters (the other), and the child was asked to touch only the target image. All images used in the practice trials were chosen randomly from the original sets of 24.

After the participant had learned the procedure, a series of test trials was administered. The task was composed of 48 trials in the first experiment, divided into two blocks of 24, and 72 trials in the second experiment, divided into two blocks of 36. For each trial, a different image matrix containing one target (snake or flower, or tilted line or vertical line) and eight distracters (the remaining image type) were presented. A trial was initiated when the experimenter judged that the participant was looking at the image, to ensure that his/her full attention was on the screen before each matrix appeared. When the first block was over, another block began. Each participant was randomly assigned to one of two block orders.

In each trial, the RT of the participant was automatically recorded as the time between the onset of the matrix presentation and image selection. The results described in the text were solely based upon analyses of the RT data collected in this manner (RTs of incorrect responses as well as extreme RT scores—defined as values more than 2 SD above or below the mean value relative to each participant’s mean RT—were excluded from the analyses).

## RESULTS

**FIGURE 1:**
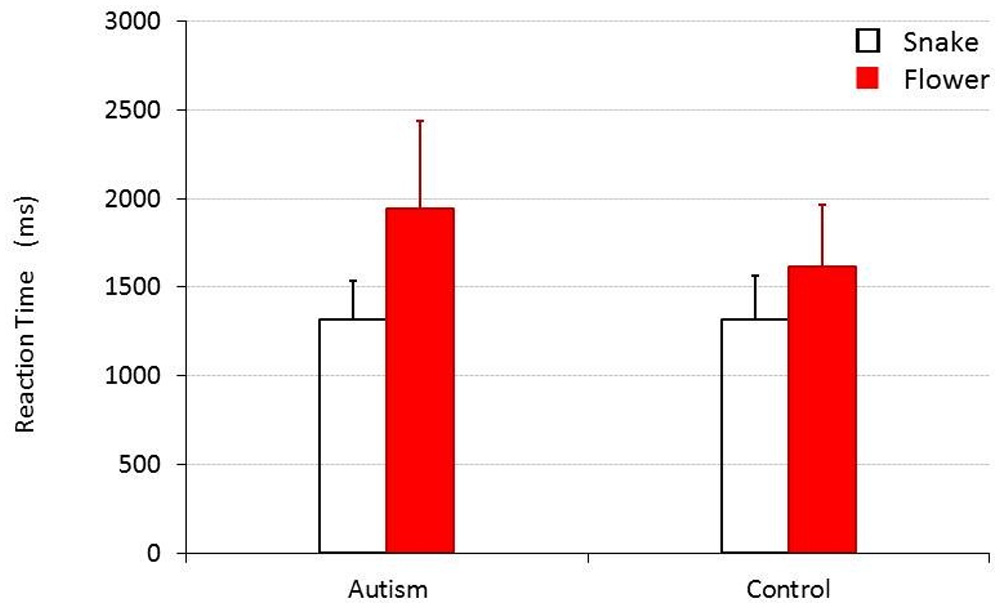
**Mean reaction time (RT) for children with and without autism to detect a snake or a flower target.** Error bars represent SDs.

The results of the experiment where snake and flower images were presented as the stimuli are shown in **Figure 1**. When the collected data were analyzed by a 2 (target image type, TARGET) × 2 (participant group; children with autism versus TD children, CHILD) repeated measure of analysis of variance, the main effect was statistically significant for TARGET [*F*_(1,30)_ = 54.275, *p* < 0.001, $\eta_{p}^{2}$ = 0.644], but not for CHILD [*F*_(1,30)_ = 2.781, *p* = 0.106, $\eta_{p}^{2}$ = 0.085]. An interaction between these factors was also significant [*F*_(1,30)_ = 7.040, *p* = 0.013, $\eta_{p}^{2}$ = 0.190]. The mean RTs (SDs) for the group of children with autism and for the control group were 1326.32 (218.36) ms and 1314.179 (247.411) ms, respectively, when they responded to a snake target, and 1961.606 (497.283) ms and 1613.036 (352.485) ms, respectively, when they responded to a flower target. *Post hoc* analyses (Bonferroni’s tests) revealed that both TD children and children with autism responded to a snake target significantly more quickly than to a flower target [*F*_(1,30)_ = 9.876, *p* = 0.004, $\eta_{p}^{2}$ = 0.248 for TD children and *F*_(1,30)_ = 57.377, *p* < 0.001, $\eta_{p}^{2}$ = 0.657 for children with autism]. There was no significant difference in how quickly TD children and children with autism responded to the snake targets [*F*_(1,30)_ = 0.022, *p* = 0.884, $\eta_{p}^{2}$ = 0.001], but that children with autism responded to flower targets significantly more slowly than TD children did [*F*_(1,30)_ = 4.933, *p* = 0.034, $\eta_{p}^{2}$ = 0.141].

**FIGURE 2:**
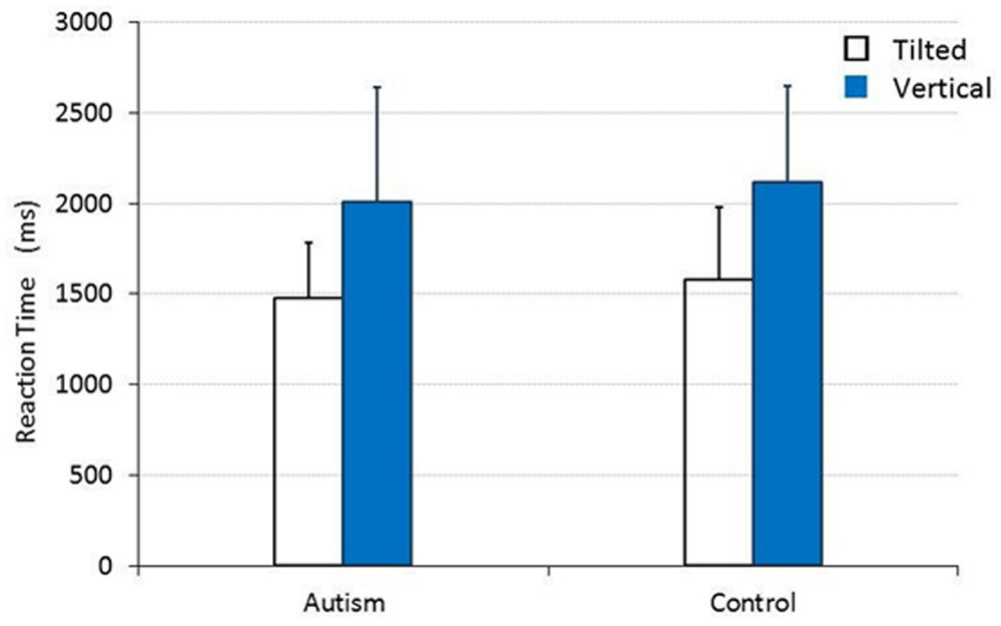
**The mean RT for children with and without autism to detect a tilted or a vertical straight line target.** Error bars represent SDs.

When vertical and tilted lines were presented, the main effect was also significant for TARGET [*F*_(1,30)_ = 49.951, *p* < 0.001, $\eta_{p}^{2}$ = 0.625], but not for CHILD [*F*_(1,30)_ = 0.445, *p* = 0.510, $\eta_{p}^{2}$ = 0.015]. As shown in **Figure 2**, there was no significant interaction between TARGET and CHILD [*F*_(1,30)_ = 0.005, *p* = 0.946, $\eta_{p}^{2}$ = 0.000]. The mean RTs (SDs) for the group of children with autism and for the control group of TD children were 1480.167 (305.700) ms and 1580.590 (401.842) ms, respectively, when they responded to a tilted line target, and 2005.947 (634.781) ms and 2116.654 (526.724) ms, respectively, when they responded to a vertical line target.

## DISCUSSION

In this study, two different experiments were conducted, each of which involved a pair of visual search tasks. In each experiment, the same two types of stimuli were presented across tasks; in one task, one image type was designated as the target, and the other item was replicated as the distractor. In the other task, the items constituting the target and the distractor were reversed. Similar experiments reported in the past have consistently shown that detection of a tilted straight line target among vertical straight line distractors is easier than vice versa, and that detection of a snake among flower distractors is easier than vice versa. This contrasting pattern for the two related tasks is known as a “search asymmetry” effect (Treisman and Souther, 1985; Treisman and Gormican, 1988) and was again confirmed by the present study in children both with and without autism.

However, in the present study, the detection of the flower targets among snake distractors was slower in children with autism than in TD children. Although children with autism took longer to detect flowers among snakes, they performed comparably to TD children in terms of RTs on all other tasks administered (detection of a tilted line among 11 vertical lines, detection of a vertical line among 11 tilted lines and detection of a snake picture among eight flower pictures). In all, the detection of the flower among the snake images by children with autism was found to be disproportionally slow.

As an explanation for the findings, two possibilities can be suggested. One of them would be that children with autism were not necessarily faster to shift attention to snakes as threatening stimuli, but that they found it harder to disengage from the threatening images than TD children. This would be consistent with the recent argument that threat-related objects are more likely to delay the disengagement of attention from the threatening stimuli than to facilitate orienting the attention (Öhman and Soares, 1993; Öhman and Mineka, 2001). As an alternative explanation, one possibility would be to assume that given the finding of even longer RTs to vertical lines as distractors in TD children, they were faster in disengaging from the snakes than all other conditions, but that this reduction of the time to disengage was not observed in children with autism. A reaction pattern toward threatening objects like that observed here in TD children is that well known as “fight or flight” responses (Shibasaki and Kawai, 2009; Masataka and Shibasaki, 2012; Shibasaki et al., 2014) provided us as part of our very basic survival predispositions, and the biological structures that support the responses also usually communicate with those that provide the ability to inhibit and control reactions (e.g., “this is not a real snake, it’s harmless, let’s go on and search for the flower”). This reasoning would lead us to explain our results as those reflecting a lack or delay in these control processes in children with autism.

Which of these explanations is correct appears to be difficult to determine on the basis of the present findings alone. If the first explanation is correct, this delayed disengagement would, in turn, somehow relate to the formation of increased phobias, one of the most characteristic clinical features of autism, which would frequently lead to social withdrawal and avoidance (Kanner, 1943; Grandin, 1996). Indeed, similar findings to our present results have been previously observed in adult anxiety patients during visual search tasks; these patients demonstrate increased distractibility by threatening stimuli (Rinck et al., 2003, 2005; Rinck and Becker, 2005). While the present study was conducted with children with autism and without any anxiety symptoms, the possibility of this explanation could be explored by comparing performance in the snake detection experiment as conducted here between such children and children with autism and with anxiety symptoms. In order to test the possibility, it would be necessary to collect some trait anxiety data from each participant, though the current experimental literature relating to the close association of autism with such emotional disturbances is surprisingly sparse: only two recent publications have attempted to evaluate fear and anxiety levels in adults with autism using fear conditioning paradigms (Bernier et al., 2005; Gaigg and Bowler, 2007), and the responses of adults with autism were found to be virtually equivalent to those of adults without autism.

In order to examine the second possibility, it would be important to conduct visual search tasks with snake images under more controlled experimental settings; it should be noted that the present experiment was performed using a touch-pad, which certainly enabled children to perform the required task more easily than if they had used a choice-button, as in the previous research (Öhman and Soares, 1993; Öhman et al., 2001; Flykt, 2005). However, it should also be noted that the use of a touch-pad makes it difficult to isolate the cognitive cause of a difference of the performance when it is recorded. Perhaps future research could involve eye tracking with little head restraint, and even quantifying pupillary dilation during visual scanning as a measure of sympathetic arousal. Also, we still have to admit the remaining possibility that the low level visual feature could explain the observed effects because we did not match the visual feature of snake stimuli versus flower stimuli. These are clearly issues that should be investigated as a next step of the study.

## Conflict of Interest Statement

The authors declare that the research was conducted in the absence of any commercial or financial relationships that could be construed as a potential conflict of interest.

## References

[B1] American Psychiatric Association. (2013). *Diagnosis and Statistical Manual of Mental Disorders* 5th Edn. Washington, DC: American Psychiatric Association. 10.1176/appi.books.9780890425596

[B2] Auyeung B. Baron-Cohen S. Wheelwright S. Allison C. (2008). The autism-spectrum quotient: children’s version (AQ-Child). *J. Autism. Dev. Disord.* 38 1230–1240 10.1007/s10803-007-0504-z 18064550

[B3] Belopolsky A. V. Devue C. Theeuwes J. (2011). Angry faces hold the eyes. *Vis. Cogn.* 19 27–36 10.1080/13506285.2010.536186

[B4] Bernier R. Dawson G. Panagiotides H. Webb S. (2005). Individuals with autism spectrum disorders show normal responses to a fear potential startle paradigm. *J. Autism. Dev. Disord.* 35 575–583 10.1007/s10803-005-0002-0 16167091

[B5] Casey B. J. Gordon C. T. Mannheim G. B. Rumsey J. M. (1993). Dysfunctional attention in autistic savants. *J. Clin. Exp. Neuropsychol.* 15 933–946 10.1080/01688639308402609 8120129

[B6] Elsabbagh M. Volein A. Holmboe K. Tucker L. Csibra G. Baron-Cohen S. (2009). Visual orienting in the early broader autism phenotype: disengagement and facilitation. *J. Child Psychol. Psychiatry* 50 637–642 10.1111/j.1469-7610.2008.02051.x 19298466 PMC3272379

[B7] Flykt A. (2005). Visual search with biological threat stimuli: accuracy, reaction times, and heart rate changes. *Emotion* 5 349–353 10.1037/1528-3542.5.3.349 16187870

[B8] Fox E. Russo R. Dutton K. (2002). Attentional bias for threat: evidence for delayed disengagement from emotional faces. *Cogn. Emot.* 16 355–379 10.1080/02699930143000527 18273395 PMC2241753

[B9] Gaigg S. B. Bowler D. M. (2007). Differential fear conditioning in Asperger’s syndrome: implications for an amygdala theory of autism. *Neuropsychology* 45 2125–2134 10.1016/j.neuropsychologia.2007.01.012 17321555

[B10] Grandin T. (1996). *Thinking in Pictures.* New York, NY: Vintage.

[B11] Hayakawa S. Kawai N. Masataka N. (2011). The influence of color on snake detection as visual search in human children. *Sci. Rep.* 1 80 10.1038/srep00080 PMC3216567 22355599

[B12] Hughes C. Russell J. (1993). Autistic children’s difficulty with mental disengagement from an object: its implications for theories of autism. *Dev. Psychol.* 29 498–510 10.1037/0012-1649.29.3.498

[B13] Isbell L. A. (2009). *The Fruit, the Tree, and the Serpent: Why We See So Well*. Cambridge, MA: Harvard University Press.

[B14] Johnson M. H. Posner M. I. Rothbart M. K. (1993). Faciliation of saccades toward a covertly attended location in early infancy. *Psychol. Sci.* 5 90–93 10.1111/j.1467-9280.1994.tb00636.x

[B15] Kanner L. (1943). Autistic disturbances of affective contact. *Nerv. Child* 2 217–250. 4880460

[B16] Landry R. Bryson S. E. (2004). Impaired disengagement of attention in young children with autism. *J. Child Psychol. Psychiatry* 45 1115–1122 10.1111/j.1469-7610.2004.00304.x 15257668

[B17] LoBue V. DeLoache J. S. (2008). Detecting the snake in the grass – Attention to fear-relevant stimuli by adults and young children. *Psychol. Sci.* 19 284–289 10.1111/j.1467-9280.2008.02081.x 18315802

[B18] LoBue V. DeLoache J. S. (2010). Superior detection of threat-relevant stimuli in infancy. *Dev. Sci.* 13 221–228 10.1111/j.1467-7687.2009.00872.x 20121878

[B19] Markram K. Markram H. (2010). The intense world theory – A unifying theory of the neurobiology of autism. *Front. Hum. Neurosci.* 4:224 10.3389/fnhum.2010.00224 PMC3010743 21191475

[B20] Masataka N. Hayakawa S. Kawai N. (2010). Human young children as well as adults demonstrate ‘superior’ rapid snake detection when typical striking posture is displayed by the snake. *PLoS ONE* 5:e15122 10.1371/journal.pone.0015122 PMC2994910 21152050

[B21] Masataka N. Shibasaki M. (2012). Premenstrual enhancement of snake detection in visual search in healthy women. *Sci. Rep.* 2 307. 10.1038/srep00307 PMC3297202 22403744

[B22] Öhman A. Flykt A. Esteves F. (2001). Emotion drives attention: detecting the snake in the grass. *J. Exp. Psychol. Gen.* 130 466–478. 11561921 10.1037/0096-3445.130.3.466

[B23] Öhman A. Mineka S. (2001). Fears, phobias, and preparedness: toward an evolved module of fear and fear learning. *Psychol. Rev.* 108 483–522. 11488376 10.1037/0033-295x.108.3.483

[B24] Öhman A. Soares J. J. F. (1993). On the automatic nature of phobic fear – Conditioned electrodermal responses to masked fear-relevant stimuli. *J. Abnorm. Psychol.* 102 121–132 10.1038/srep00307 8436688

[B25] Posner M. I. Dehaene S. (1994). Attentional networks. *Trend Neurosci.* 17 75–79 10.1037/0021-843X.114.2.235 7512772

[B26] Rinck M. Becker E. S. (2005). A comparison of attentional biases and memory biases in women with social phobia and major depression. *J. Abnorm. Psychol.* 114 62–74 10.1037/0096-3445.130.3.466 15709813

[B27] Rinck M. Becker E. S. Kellermann J. Roth W. S. (2003). Selective attention in anxiety: distraction and enhancement in visual search? *Depress. Anxiety* 18 18–28 10.1037/0033-295X.108.3.483 12900949

[B28] Rinck M. Reinecke A. Ellwart T. Heuer K. Becker E. S. (2005). Speeded detection and increased distraction in fear of spiders: evidence from eye movement. *J. Abnorm. Psychol.* 114 235–248 10.1037/0021-843X.102.1.121 15869354

[B29] Shibasaki M. Isomura T. Masataka N. (2014). Viewing images of snakes accelerates making judgments of their colour in humans: red snake effect as an instance of ‘emotional Stroop facilitation’. *R. Soc. Open Sci.* 1 140066 10.1037/0021-843X.114.1.62 PMC4448842 26064551

[B30] Shibasaki M. Kawai N. (2009). Rapid detection of snakes by Japanese monkeys (*Macaca fuscata*): an evolutionarily predisposed visual system. *J. Comp. Psychol.* 123 131–135 10.1002/da.10105 19450020

[B31] Treisman A. Gormican S. (1988). Feature analysis in early vision: evidence form search aymmetries. *Psychol. Rev.* 95 12–48 10.1098/rsos.140066 3353475

[B32] Treisman A. Souther J. (1985). Search asymmetry: a diagnostic for preattentive processing of separable features. *J. Exp. Psychol. Gen.* 114 285–310 10.1037/a0015095 3161978

[B33] Van Der Geest J. N. Kemner J. N. Camfferman G. Verbaten M. N. Van Engeland H. (2001). Eye movements, visual attention, and autism: a saccadic reaction time study using the gap and overlap paradigm. *Behav. Psychiatry* 50 614–619 10.1016/0166-2236(94)90078-7 11690597

[B34] Yorzinski J. I. Penknus M. J. Platt M. L. Coss R. G. (2014). Dangerous animals capture attention in humans. *Evol. Psychol.* 12 534–548 10.1037/0033-295X.95.1.15 25299991 PMC10480850

